# Thalamic dopamine D2-receptor availability in schizophrenia: a study on antipsychotic-naive patients with first-episode psychosis and a meta-analysis

**DOI:** 10.1038/s41380-021-01349-x

**Published:** 2021-11-10

**Authors:** Pontus Plavén-Sigray, Pauliina Ikonen Victorsson, Alexander Santillo, Granville J. Matheson, Maria Lee, Karin Collste, Helena Fatouros-Bergman, Carl M. Sellgren, Sophie Erhardt, Ingrid Agartz, Christer Halldin, Lars Farde, Simon Cervenka

**Affiliations:** 1 Centre for Psychiatry Research, Department of Clinical Neuroscience, Karolinska Institutet and Stockholm Health Care Services, Region Stockholm, Stockholm, Sweden; 2 Neurobiology Research Unit, Copenhagen University Hospital, Rigshospitalet, Copenhagen Denmark; 3 Clinical Memory Research Unit, Department of Clinical Sciences, Lund University, Malmö, Sweden; 4 Molecular Imaging and Neuropathology Division, Columbia University, New York, NY USA; 5 Department of Biostatistics, Columbia University, New York, NY USA; 6 Department of Physiology and Pharmacology, Karolinska Institutet, Stockholm, Sweden; 7 Norwegian Centre for Mental Disorders Research (NORMENT), Institute of Clinical Medicine, University of Oslo, Oslo, Norway; 8 Department of Psychiatric Research, Diakonhjemmet Hospital, Oslo, Norway; 9 Department of Neuroscience, Psychiatry, Uppsala University, Uppsala, Sweden

**Keywords:** Schizophrenia, Biochemistry

## Abstract

Pharmacological and genetic evidence support a role for an involvement of the dopamine D2-receptor (D2-R) in the pathophysiology of schizophrenia. Previous molecular imaging studies have suggested lower levels of D2-R in thalamus, but results are inconclusive. The objective of the present study was to use improved methodology to compare D2-R density in whole thalamus and thalamic subregions between first-episode psychosis patients and healthy controls. Differences in thalamocortical connectivity was explored based on the D2-R results. 19 antipsychotic-naive first-episode psychosis patients and 19 age- and sex-matched healthy controls were examined using high-resolution Positron Emission Tomography (PET) and the high-affinity D2-R radioligand [^11^C]FLB457. The main outcome was D2-R binding potential (BP_ND_) in thalamus, and it was predicted that patients would have lower binding. Diffusion tensor imaging (DTI) was performed in a subgroup of 11 patients and 15 controls. D2-R binding in whole thalamus was lower in patients compared with controls (Cohen’s dz = −0.479, *p* = 0.026, Bayes Factor (BF) > 4). Among subregions, lower BP_ND_ was observed in the ROI representing thalamic connectivity to the frontal cortex (Cohen’s dz = −0.527, *p* = 0.017, BF > 6). A meta-analysis, including the sample of this study, confirmed significantly lower thalamic D2-R availability in patients. Exploratory analyses suggested that patients had lower fractional anisotropy values compared with controls (Cohen’s *d* = −0.692, *p* = 0.036) in the inferior thalamic radiation. The findings support the hypothesis of a dysregulation of thalamic dopaminergic neurotransmission in schizophrenia, and it is hypothesized that this could underlie a disturbance of thalamocortical connectivity.

## Introduction

The dopamine system has for several decades been of central importance for research on the pathophysiology and treatment of schizophrenia. Well over one hundred molecular imaging studies have investigated the in vivo effects of antipsychotic treatments, showing that all currently licensed antipsychotic drugs block dopamine D2 receptors (D2-R) [1, 2]. Moreover, a causal role for D2-R in the disease mechanism of schizophrenia has been suggested by genetic studies [3]. Molecular imaging studies have shown a slight increase in striatal D2-R in psychosis and schizophrenia patients compared with controls, although this was not significant in the subsample of antipsychotic-naive first-episode patients [4]. However several other brain regions have been considered more important in generating the diversity of symptoms observed in schizophrenia. Further investigations of dopaminergic function outside of the striatum is therefore needed in order to understand the pathophysiology of the disorder.

A region of key interest in schizophrenia is the thalamus, which is richly interconnected to the cortex, and plays a central role in coordinating signaling both to and in between cortical regions [5, 6]. In a series of positron emission tomography (PET) studies using the high-affinity D2-R radioligands [^11^C]FLB 457 and [^18^F]Fallypride, which were developed for low-density extrastriatal regions [7, 8], D2-R binding has shown to be lower in thalamus in patients with psychosis compared with controls [9–12]. However, no differences, or increases have also been observed using these radioligands [13–16]. A meta-analysis, which also included studies using other radioligands, showed lower thalamic D2-R binding in patients (*d* = −0.32), but the difference was not statistically significant [17]. Importantly, the analysis included several studies with patients with previous exposure to medication and in later disease stages, and some of the studies used radioligands with low signal-to-noise for quantifying D2-R in extrastriatal brain regions. Hence, additional studies are needed to confirm if there indeed is a decrease of D2-R in thalamus in schizophrenia, preferably using high-affinity radioligands in samples not affected by antipsychotic drug treatment and other confounders stemming from chronical illness.

The thalamic complex is functionally heterogenous, being part of parallel circuits with distinct physiological implications. Neuropathological studies in schizophrenia have primarily shown a significant reduction in the number of neurons in the thalamic mediodorsal and pulvinar subregions [18–20]. In some of the previous PET studies, low D2-R binding was found specifically in medial [9–12] and posterior subregions of the thalamus [10]. However, the low resolution of the PET systems used in these studies and the diversity in region of interest (ROI) definition limit the conclusions that can be drawn. Additionally, none of the previous studies based the regional analysis on underlying functional anatomy.

The primary aims of this study were to (1) confirm previous observations of lower D2-R binding in the thalamus in psychosis and schizophrenia patients, (2) extend the subregional D2-R analysis using improved methodology, and (3) based on subregional D2-R differences, investigate aberrations in thalamo-cortical connectivity in an exploratory manner. For these purposes, we used high-resolution PET and the high-affinity radioligand [^11^C]FLB457 to examine 19 drug-naive first-episode psychosis patients and 19 matched healthy control subjects. Thalamic subregions were definied using a connectivity-based atlas. An exploratory diffusion tensor imaging (DTI) analysis of thalamic structural connectivity was performed based on the PET results for a subgroup of individuals.

## Subjects and methods

### Patients and healthy control subjects

The study was approved by the Stockholm Regional Ethics Committee (Dnr 2010/879-31/1) and the Radiation and Safety Committee at the Karolinska University Hospital. All subjects provided written consent after receiving a complete description of the study, according to the Helsinki declaration. Patients with first-episode psychosis, as defined by first contact with psychiatric services due to psychotic symptoms, were recruited from outpatient clinics and psychiatric wards in the Stockholm region. At time of recruitment and during their participation in the study, all patients were fully naive to antipsychotic drugs. The final patient sample consisted of 19 subjects (11 males, 8 females, mean (SD) age 29.3 (6.3)). Patients fulfilled criteria for the following Diagnostic and Statistical Manual of Mental Disorders IV diagnoses at inclusion: schizophrenia (*N* = 6), schizophreniform disorder (*N* = 5), psychotic disorder NOS (*N* = 5), and delusional disorder (*N* = 3), as assessed using SCID-I. Symptoms were rated using the Positive And Negative Syndrome Scale (PANSS).

Nineteen healthy control subjects (11 males, 8 females, mean (SD) age 29.2 (5.9)) were recruited by advertisement to match against a corresponding patient of the same age (±2 years) and the same sex. Control subjects were healthy according to medical history, clinical examination, routine laboratory blood test as well as a brain magnetic resonance imaging (MRI) examination.

Two patients and two healthy control subjects used nicotine on a daily basis. Eight patients received anxiolytics (benzodiazepine (5 patients, oxazepam, mean = 11 mg, range 5–15 mg) and/or antihistamines (5 patients)) during their participation in the PET study. For exclusion criteria see Supplementary information.

### MRI, DTI examinations, and ROI delineation

Structural MRI data was obtained using a 3-T General Electric Discovery MR750 system (GE, Milwaukee, WI, USA). T1-weighted images were used for ROI delineation, and T2-weighted FLAIR images were assessed for pathology by a neuroradiologist. One control subject was examined on a 1.5T GE Signa system (Milwaukee, WI) instead of the 3 T-system.

Diffusion-weighted data were collected for a subset of 11 patients and 15 control subjects with a spin-echo sequence in the axial plane with diffusion gradients in 60 directions and *b*-values of 1000 s/mm^2^. Images were acquired with a field of view of 240 mm and a slice thickness of 290 mm, with a voxel size of 1.875 × 1.875 × 2.9 mm.

**Fig. 1:**
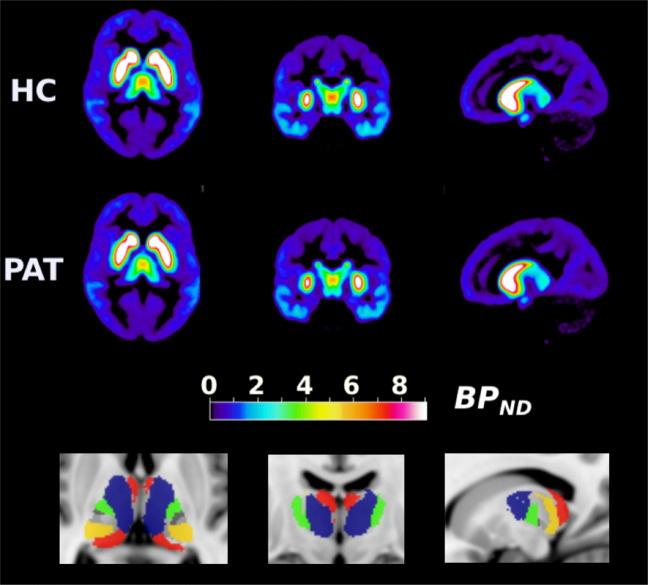
Average BP_ND_ values and thalamic ROIs. Upper panel: average BP_ND_ image values for first-episode psychosis patients and matched control subjects. Lower panel: ROIs for thalamic subregions shown on a template T1 MR image, based on their connectivity to cortical brain regions. Blue = THA-PFC (projections to prefrontal cortex), red = THA-TC (temporal cortex), gray = THA-M1 (primary motor cortex), green = THA-PreMC (premotor cortex), orange = THA-PPC (posterior parietal cortex).

Thalamic ROIs were obtained using the FMRIB FSL v.5.0.7 software [21]. For whole thalamus ROI, the FSL Harvard-Oxford subcortical atlas was used with a 50% probability threshold. For thalamic subregionals delineation, the Oxford Thalamic Connectivity Atlas [22] (probability thresholding at 50%) was used to extract the following ROIs: (1) Anterior part of the mediodorsal nucleus and ventral anterior nuclei with DTI projections to the prefrontal cortex (THA-PFC ROI); (2) the most medial part of the mediodorsal nucleus, parts of the anterior complex and medial and inferior pulvinar that projects to the temporal cortex (THA-TC); (3) ventral posterolateral nucleus which projects to primary motor cortex (THA-M1); (4) ventral lateral nucleus and the ventral anterior nucleus which project to premotor areas (THA-PreMC); and (5) anterior parts of the pulvinar that project to posterior parietal cortex (THA-PPC) (Fig. 1). For additional details see Supplementary information. Since we had no hypothesis of hemispheric differences, right and left hemisphere ROIs were pooled to increase count statistics.

A cerebellum mask was also extracted using the maximum probability FSL MNI FNIRT atlas. The mask was then trimmed to exclude voxels close to the vermis, the cortex, and the most inferior part of the PET image, as has been described previously [23]. Subsequently, all ROIs were warped back into the individual T1-image space.

### PET examinations and data quantification

All patients and all healthy controls were examined using the high-resolution research tomograph (Siemens Molecular Imaging, Knoxville, TN, USA) at the Karolinska Institutet PET center. A drug toxicology screen was performed prior to the PET examination. [^11^C]FLB 457 was prepared as described previously [24] and injected into the antecubital vein as a rapid bolus. Mean (SD) injected activity was 425.95 (47.98) MBq, mean (SD) specific activity was 374 (203) Gbq/micromol, and mean injected mass was 0.4–0.7 microg. All emission scans were 90 min long. Non-invasive Logan Graphical Analysis [25] with cerebellum as reference region was used to obtain BP_ND_ values in all thalamic ROIs. All quantitative analyses were performed using the “kinfitr” package in R [26]. For additional details, see Supplementary information.

### Exploratory DTI analysis

The thalamus and its subregions (i.e., as defined by Oxford Thalamic Connectivity Atlas, see above), connect to cortical regions by axons gathered into white matter tracts, radiations. Based on the results of the PET analyses of thalamic subregions we chose to study the two major radiations leading to and from the TH-PFC ROI, the anterior thalamic radiation (ATR) and inferior thalamic radiation (ITR) [22, 27]. This was done by manual dissection according to existing protocols [28, 29] on whole brain tractography with a fractional anisotropy threshold of 0.2 and an angle threshold of 30°, using ExploreDTI [30]. See Supplementary information and Supplementary Fig. 1 for a detailed description and illustration. Motion and eddy current correction of the data was performed using ElastiX [31]. Diffusion-weighted sequences were only available for a subset of the study subjects (11 patients and 15 healthy controls).

### Statistical analysis

In this study, the statistical analyses and procedures related to analysis of PET data (whole thalamus and subthalamic analyses) were pre-registered a priori and are hence considered to be confirmatory analyses. The DTI analyses were considered to be exploratory in nature, with the aim of generating new hypotheses regarding connectivity changes associated with an aberrant thalamic D2-R system. Since previously reported effect sizes of patient-control differences in thalamic D2-R availability have been small to medium in size [17], we elected to form directed (i.e. one-sided) a priori hypotheses in this study, in conjunction with paired matching of patients and controls, in order to maximize the power of the tests. This entails us making more specific predictions about the effect, which affords us a higher degree of sensitivity, assuming that these predictions were appropriate. In this way, we are leveraging the findings of prior research to improve the power of our research design while minimizing the number of participants who must be exposed to radioactivity to test these hypotheses. The preregistration protocol (https://osf.io/nhr3w/) further outlines the a priori predictions and the rationale behind the chosen statistical tests.

To examine patient-control differences in [^11^C]FLB 457 BP_ND_, we performed a frequentist paired-sample *t*-test using a one-sided hypothesis, i.e. expecting patients to have lower [^11^C]FLB 457 BP_ND_ (specified a priori in the preregistration document). We examined the whole thalamus as well as thalamic subregions (setting *α* = 0.05). We also applied a Bayesian paired-sample *t*-test, in which we made use not only of the expected direction of the effect, but also the expected magnitude observed in previous studies. This was done using a meta-analytical effect size on D2-R patient-control differences in thalamus and its uncertainty as a prior over the research hypothesis, which we compared to the null hypothesis of no difference [32] (see Supplementary information for details). This approach, also specified a priori in the preregistration document, allows us to make even more specific predictions compared to the frequentist approach, by taking previously available information into account when shaping the research hypothesis. By using the Bayes Factor (BF) we then evaluated whether the collected data are more consistent with the hypothesis of decreases in thalamic D2-R binding in psychosis and schizophrenia patients (H_1_), or with the null hypothesis of no effect (H_0_). A BF in favor of H1 relative to H0 (BF_H1:H0_) above 3 is commonly interpreted as moderate evidence in favor of the alternative hypothesis, and in the present study would be viewed as support for a successful replication of the previous findings of lower patient binding. A BF in favor of H0 relative to H1 (BF_H0:H1_) above 3 is commonly interpreted as moderate evidence in favor of the null hypothesis, indicating that no difference in binding between patients and controls gained support in our study [33].

To correct for multiple comparisons in the subregional analysis, a permutation procedure was employed [34] to estimate the average increase in type I error, while taking the dependency between the five ROIs into account, resulting in a family-wise error corrected *α* of 0.0356 (see Supplementary information for additional details).

The statistical analyses presented in the main text deviates from the preregistration in one important manner. When defining the alternative hypothesis for the BF test of whole-thalamus difference above, we opted to also include the study by Talvik et al. [12], Yasuno et al., Slifstein et al. [13], and Veselinovic et al. [15] in addition to the studies reported in the Kambeitz et al. meta-analysis [17]. A systematic literature search was performed dating from January 1st 2013 to March 16th 2021 to make sure no additional studies were overlooked, allowing us to shape an H_1_ informed by all previous published literature (see Supplementary information and Supplementary Fig. 2 for additional details). For full transparency the results from the BF-analysis carried out as written in the preregistration are also reported in the Supplementary information (the overall conclusion does not differ between these two analyses). In the results section, we consequently opted to present meta-analytic results on thalamic D2-R availability differences between patients with psychotic disorders and healthy controls, combining the results from this study with the previous literature (based on the literature search above). One analysis was conducted using all published studies, similar to Kambeitz et al., and we also performed a separate analysis including only studies using either of the two high-affinity radioligands [^18^F]Fallypride or [^11^C]FLB457, which have higher sensitivity for low-density D2-R regions than all other radioligands used. A further addition to the preregistration protocol is that frequentist and BF two-sample *t*-tests were also performed (presented in the supplementary tables), in addition to the paired *t*-tests. All statistical analyses were carried out using R (v. 3.5.1 “Feather Spray”) or JASP (v. 0.10.2).

For the DTI analyses, the literature has shown both increases and decreases of fractional anisotropy in psychosis and schizophrenia, although most studies appear to report increases [35, 36]. Since the analyses were exploratory, we opted to make use of a two-sided *t*-test, without any corrections for multiple comparisons. *P*-values from these analyses are hence to be considered only as continuous assessments of indirect evidence against the null, and not of confirmatory nature [37]. Since DTI data was only available for a subset of the subjects, matching was not possible and the analysis was unpaired. For information on patient-control matching see Supplementary information.

## Results

Patients were moderately ill, with average PANSS and CGI scores of 65.6 and 4.5 respectively, and the duration of illness was 12.1 months. Additional demographic, radiochemical, and clinical variables for patients and healthy controls are presented in Table 1.

**Table 1:** Descriptive demographic, radiochemical, and clinical data for patients and healthy control subjects.

	Controls		Patients		
	Mean (SD)	Min–max	Mean (SD)	Min–max	*p*
Age (years)	29.2 (5.9)	20–43	29.3 (6.3)	18–42	0.75
Body mass index (kg/m^2^)	24.7 (3.1) (*N* = 16)	18.5–30	23.5 (2.4)	20–31	0.33
Education (years)	15.2 (2.2) (*N* = 17)	12–20	15.2 (3.3) (*N* = 17)	10–22	0.77
Injected activity (MBq)	431.4 (47.1)	317–480	420.5 (49.5)	292–480	0.48
Molar Mass (GBq/mmol)	285.5 (125.3)	122–543	462.1 (228.6)	117–955	0.01
Injected mass (microg)	0.7 (0.4)	0.27–1.31	0.4 (0.2)	0.17–0.93	0.01
Cerebellum SUV total	6315 (1924)	3290–9974	5830 (2227)	2569–10297	0.35
Cerebellum SUV 25–85 min	55 (17)	30–80	51 (20)	22–96	0.37
Duration of illness (months)			12.1 (19.5)	1–84	
CGI			4.5 (1.0)	3–6	
PANSS-total			65.6 (17.4)	39–97	
PANSS-global			33.6 (9.7)	18–54	
PANSS-positive			17.7 (4.2)	11–24	
PANSS-negative			14.4 (6.2)	7–28	

*SUV* Standardized uptake values, *CGI* Clinical Global Impressions Scale, *PANSS* Positive and Negative Syndrome Scale.

### Analysis of D2-R availability in the whole thalamus

**Fig. 2:**
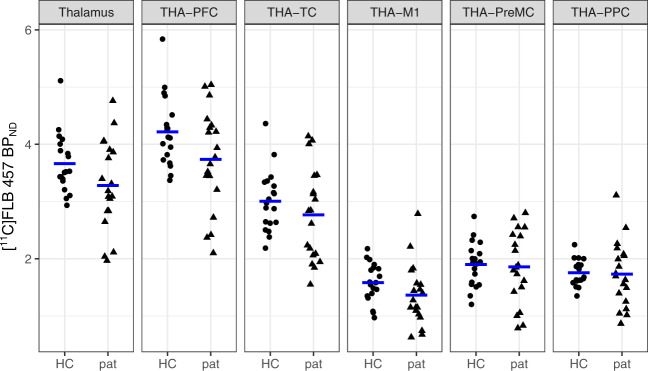
Patient-control differences in [^11^C]FLB 457 BP_ND_ in the whole thalamus and subthalamic regions. There was a significant difference between groups in the whole thalamus as well as the subregion corresponding to prefrontal cortex connectivity.

The frequentist paired *t*-test showed a significant difference between patients and healthy controls in [^11^C]FLB 457 BP_ND_ in the whole-thalamus ROI, with patients having lower binding compared with healthy controls (Table 2 and Fig. 2). The BF_H1:H0_ from the Bayesian paired *t*-test indicated that the data was ~5 times more likely to have originated under the alternative hypothesis compared to the null hypothesis of no difference.

**Table 2:** Results from frequentist and Bayesian paired-sample *t*-tests showing patient-control differences in [^11^C]FLB 457 BP_ND_ in thalamus.

Region	Mean BP_ND_ (SD) controls	Mean BP_ND_ (SD) patients	Mean BP_ND_ difference (SD) (pat-HC)	Lower–Upper 95% CI	Mean %-difference (pat-HC)	*p*	BF H1:H0	Cohen’s dz
Whole thalamus	3.66 (0.52)	3.28 (0.79)	−0.38 (0.80)	−0.77 to 0.00	−10	0.03	4.7	0.48
Thalamus-prefrontal Cx	4.22 (0.61)	3.74 (0.88)	−0.48 (0.91)	−0.92 to −0.04	−11	0.02	5.8	0.53
Thalamus-temporal Cx	1.47 (0.44)	1.14 (0.41)	−0.24 (0.82)	−0.64 to 0.16	−22	0.11	1.4	0.29
Thalamus-primary motor Cx	1.58 (0.34)	1.36 (0.53)	−0.22 (0.69)	−0.55 to 0.11	−14	0.09	1.6	0.32
Thalamus-premotor Cx	1.90 (0.39)	1.85 (0.64)	−0.04 (0.73)	−0.40 to 0.31	−3	0.40	0.5	0.06
Thalamus-posterior parietal Cx	1.75 (0.22)	1.73 (0.57)	−0.02 (0.56)	−0.29 to 0.25	−1	0.43	0.5	0.04

For informative and default priors for the Bayes Factor (BF) see methods. All *t*-tests were one-sided expecting lower levels in patients. BF H1:H0 = support in data in favor of the alternative hypothesis over the null hypothesis as quantified using Bayes Factor.

*Cx* cortex.

### Meta-analyses

**Fig. 3:**
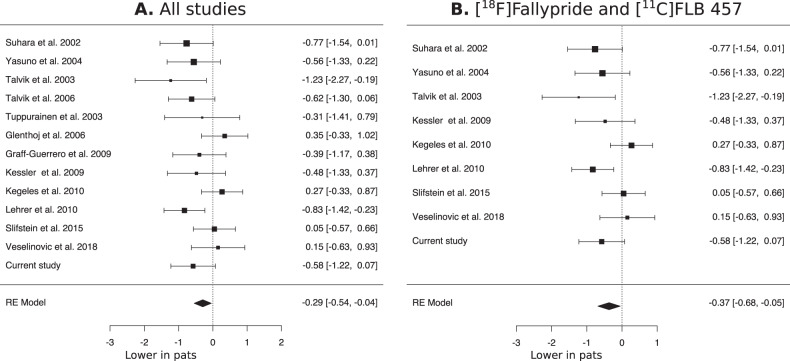
Meta-analyses of thalamic D2-R availability differences between patients and controls. **A** Patients show significantly overall lower D2-R levels in whole thalamus compared with healthy control subjects. **B** Meta-analysis only including studies which used the two high-affinity radioligands [^11^C]FLB457 and [^18^F]Fallypride. Additional meta-analyses excluding studies with partially overlapping samples, and funnel plots can be found in the supplementary information.

When combining the results from this study with previous literature on D2-R availability differences in the whole thalamus, the meta-analysis showed a significant overall lower binding in patients with psychotic disorders compared with healthy controls (pooled effect size −0.29, Fig. 3a). This was also the case when including only studies performed using the two high-affinity radioliogands [^18^F]Fallypride and [^11^C]FLB457, with a slightly larger pooled effect size of −0.37 (Fig. 3b).

### Analysis of D2-R availability in thalamic subregions

Among the thalamic subregions defined by the Oxford Thalamic Connectivity atlas, the thalamus-PFC ROI showed the largest effect (Table 2), with BF_H1:H0_ indicating that there was 6 times more support for the alternative hypothesis compared to the null hypothesis (Table 2 and Fig. 2). The difference was also significant with a *p*-value (0.017) below the family-wise error rate corrected alpha threshold. No other subregion in the thalamus showed significant differences, and support for either hypothesis was inconclusive according to the Bayesian paired *t*-tests (BF_H1:H0_ and BF_H0:H1_ < 3).

### DTI analysis

The final objective of the study was to explore possible differences in structural thalamocortical connectivity using DTI, with fractional anisotropy of the white matter tracts as the parameter of interest. For the ITR, data were skewed and the non-parametric Mann–Whitney *U*-test was used when comparing patients and healthy subjects. Patients showed lower fractional anisotropy values (mean = 0.375, 0.017 SD) compared with healthy control subjects (mean = 0.389, 0.020 SD, Cohen’s *d* = −0.692, *p* = 0.036) in the ITR, whereas fractional anisotropy values of the ATR showed very similar values in patients (mean = 0.423, 0.0145 SD) and controls (mean = 0.426, 0.023 SD, Cohen’s *d* = 0.128, *p* = 0.75).

### PANSS correlations

Associations were explored between the PANSS total, PANSS positive and PANSS negative scores and BP_ND_ in the whole thalamus and in the Thalamus-PFC subregion, finding no significant correlations (see Supplementary information).

### Robustness checks

After observing a significant difference between patients and controls in injected mass (Table 1) we examined the association between injected mass, specific molar activity, and [^11^C]FLB457 BP_ND_, finding no association (see Supplementary information). No significant difference was observed between the cerebellar SUV of patients and controls (Supplementary Table 1). There was also no difference in thalamic volumes between patients and controls (Supplementary Table 2).

Two-sample (unpaired) *p*-values for the BP_ND_ patient-control comparisons in whole thalamus and thalamic subregions showed similar results as in the main analysis (Supplementary Table 3). We also re-ran the main analyses after having excluded four participants with partially incomplete or missing data, finding somewhat higher *p*-values but unchanged effect sizes (Supplementary Table 4).

We performed a set of robustness-checks over the alternative hypotheses in the BF *t*-tests. These also provided evidence in favor of alternative hypothesis of lower D2-R availability in patients (Supplementary Table 5, Supplementary Fig. 3, and Supplementary information).

Funnel plots for the meta-analyses are shown in Supplementary Fig. 4, revealing no apparent publication bias.

Finally, we performed an additional meta-analysis excluding Talvik et al. [38] and Yasuno et al. [10], since there was some overlap in the sample between these studies and Talvik et al. [12] and Suhara et al. [14], respectively. This exclusion did not change the overall finding of lower thalamic D2-R availability in patients compared with healthy controls (Supplementary Fig. 5).

## Discussion

In this PET study examining thalamic D2-R in a sample of only drug-naive psychosis patients compared to healthy controls we observed lower binding in patients. This is the first study investigating thalamic subregions in psychosis patients using high-resolution PET. By employing a connectivity-based atlas we identified lower D2-R levels in a thalamic subregion with projections to the frontal cortex. In a subgroup of patients, we observed a tentative result of lower fractional anisotropy values compared with controls in the ITR, which predominantly projects to and from the thalamus and the orbitofrontal, temporal and insular cortex. Taken together, the PET results strengthen the evidence for changes in dopaminergic neurotransmission in the thalamus as a part of the pathophysiology in schizophrenia. Based on the exploratory DTI analyses, we further hypothesize that these may underlie aberrant connectivity between regions shown to be of importance for the pathophysiology of the disorder. Notably, an increase or no change in striatal D2-R has been observed in schizophrenia and psychosis patients [4], suggestive of regionally specific alterations of dopaminergic transmission.

Previous studies investigating D2-R in thalamus have yielded inconsistent results. Two early studies using [^11^C]FLB457 in drug-naive samples showed lower D2-R binding in the thalamus [10, 12]. The sample in Talvik et al. [12] was then extended to 18 patients in a study using the medium affinity radioligand [^11^C]raclopride showing lower D2-R in the right thalamus compared with controls [38]. A more recent study using [^11^C]FLB457 in a combination of drug naive and drug free patient showed no difference in thalamic binding [13]. In two studies, single photon emission computer tomography (SPECT) and the radioligand [^123^I]epidepride was used in 25 and 6 antipsychotic-naive patients respectively, showing no significant difference in binding between patients and controls [39, 40]. Two studies that employed PET and the radioligand [^18^F] Fallypride in a combination of drug-naive and drug-free patients, showed lower BP_ND_ values medial thalamic subregions [9, 11] whereas one study showed an increase in whole thalamus [16] and yet another one found no difference [15]. Finally, two studies using the agonist and D3-R preferring radioligand [^11^C]PHNO in 13 drug-free and 12 drug-naive patients respectively showed no significant difference compared with controls [41, 42].

In these studies, sample sizes have generally been small, and several studies have had methodological limitations. SPECT has much lower spatial resolution than PET, a factor which is critical when examining smaller structures in the brain. Of the radioligands used, [^18^F]Fallypride and [^11^C]FLB457 are high-affinity tracers, while [^11^C]raclopride shows low specific binding and poor convergent validity in thalamus [43, 44]. Importantly, several studies included patients previously treated with antipsychotics [9, 11, 13, 15, 16, 41], which has been shown to result in increased D2-R levels in post-mortem and in vivo studies [45, 46]. Thus, medication effects may have masked “true” reductions in baseline D2-R in some previous studies. In the present study we exclusively included antipsychotic-naive patients, and used high-resolution PET in combination with a high-affinity radioligand for D2-R. To inform our analysis, we used all previously published data on differences between healthy controls and drug-free patients with psychotic disorders or schizophrenia, confirming support in favor of decreased D2-R availability in patients. Finally, by adding the results from this study to the previous studies in a meta-analysis, we show an overall significantly lower D2-R availability in patients (Fig. 3) (despite possible medication effects in previous studies), which is major step towards resolving the uncertainty in the previous literature.

It should be noted however that the overlap between the patient and healthy control group is large in both the current study and in the meta-analyses, with the 95% CIs being both compatible with a medium sized, as well as a negligible group-difference. This suggests that a subgroup of patients may differ in thalamic D2-R receptor levels compared with healthy participants, but that there will also be patients in the population showing little to no aberration. Further studies in larger samples are needed to identify such subgroups.

The thalamic complex includes a large number of cytoarchitectonically distinct nuclei, each being components of circuits with distinct physiological and functional implications [22, 47]. Whereas previous studies have explored arbitrarily defined thalamic subregions, we used an atlas of thalamic subregions based on DTI connectivity data [22], which in combination with high-resolution PET provides an increase in functional-anatomical specificity. Our results are consistent with aberrant dopaminergic functioning in the subregion relaying to the prefrontal cortex. The frontal cortex is believed to play an important role in the pathology behind psychotic disorder, and has been centrally implicated in both positive, negative and cognitive symptoms of schizophrenia [48–50].

From a functional neuroanatomical perspective, the frontal cortex is a large and heterogenous area. Using DTI we attempted to be more specific, assuming that altered receptor levels are related to alterations in structure that is reflected in the white matter structure and DTI metrics. Our combined PET (pointing at prefrontal cortex) and DTI results suggest changes in DA neurotransmission in thalamocortical circuits involving more specific parts of the prefrontal cortex, namely the orbitofrontal cortex. It also allows us to hypothesize an involvement of the frontal insular cortex, which is included in the thalamus-PFC ROI used [22]. In contrast, we did not observe any differences in the ATR, which has been implicated in other DTI studies on antipsychotic-naive first-episode psychosis patients [51]. Orbitofrontal cortex and insula have both shown structural changes in antipsychotic-naive schizophrenia subjects as measured with voxel-based morphometry [52, 53] and are central to in reward processing and self-awareness, two functions that are thought to be perturbed in schizophrenia [50, 54].

A possible factor underlying the observed changes in dopamine receptor levels may be loss of synaptic connections. Post-mortem studies have shown widespread loss of synaptic structures, as well as reduction of synaptic markers in schizophrenia [55, 56]. Recently a PET study employing the radioligand [^11^C]UCB-J, which binds to the synaptic vesicle glycoprotein 2 A showed widespread lower binding in patients compared with controls, indicating lower synaptic density in cortical regions as well as the thalamus [57]. Further, mechanistic data based on patient-derived cellular models indicate increased microglial synapse elimination in schizophrenia and link the schizophrenia risk locus containing the complement component 4 isotype genes to excessive synapse elimination [58]. This motivates further investigations into the relationship between synaptic loss and connectivity in thalamocortical projections, and dopaminergic function in schizophrenia.

A potential limitation of the present analysis is that a series of studies have shown that a small amount of specific [^11^C]FLB457 binding is present in the cerebellum for most subjects [59–61], such that a more ideal quantification of binding would be to measure radioactivity in arterial blood to use as an arterial input function. Although non-significant differences between the cerebellar SUV of patients and controls were observed, cerebellar binding cannot be fully excluded as a potential confounder. Another possible confounder is that of partial volume effects, meaning that smaller volumes in patients could lead to increased loss of signal, resulting in false low BP_ND_ values. However, volumes for whole thalamus as well as thalamic subregions were numerically higher in this specific patient sample, making this explanation unlikely. Finally, 5 patients had received low doses of bensodiazepines at the time of PET which may have influenced the results [62]. Apart from the limitation constituted by the modest sample size, there are technical constraints in the DTI analysis such as the low and anisotropic resolution. In addition, tractography of the ITR suffers from its orbitofrontal course which is a distortion-prone region in MRI. Our anatomical description is however in line with that of other researchers using DTI [22, 28] and the animal literature [27].

In conclusion, our findings support the hypothesis of a dysregulation of thalamic dopaminergic neurotransmission in schizophrenia. Based on the results, we hypothesize that such dopaminergic aberrations could in turn could underlie a disturbance of specific thalamocortical circuits. This should be investigated in future studies, including a larger sample of drug-naive patients examined with DTI.

## Supplementary information

Supplemental Material

## Data Availability

Due to institutional restrictions, the data of this project cannot be shared on a public repository. Instead, the data can be made available upon request on a case by case basis as allowed by the legislation and ethical permits. Requests for access can be made to the Karolinska Institutet’s Research Data Office at rdo@ki.se. The R analysis code and preregistration protocol can be found on https://osf.io/nhr3w/.
