# Supplementary material for: Thalamic dopamine D2-receptor availability in schizophrenia: a study on antipsychotic-naive patients with first-episode psychosis and a meta-analysis
Source: Mol Psychiatry. 2021 Nov 10;27(2):1233–40. doi: 10.1038/s41380-021-01349-x (PMC9054658; doi:10.1038/s41380-021-01349-x)
Supplement: Supplementary file 1 — Supplemental Material [file 41380_2021_1349_MOESM1_ESM.docx]

# ­­­­­Supplementary information

Thalamic dopamine D2-receptor availability in schizophrenia: a study on antipsychotic-naive patients with first-episode psychosis and a meta-analysis

### Patients and healthy controls

Exclusion criteria for patients were neurologic or severe somatic illness, and current use or history of abuse of alcohol or illegal drugs (including cannabis), as assessed using clinical interview as well as AUDIT and DUDIT questionnaires ^1,2^. Exclusion criteria for controls were previous or current psychiatric or somatic illness, previous or current use of alcohol or illegal drugs (assessed using clinical interview, AUDIT and DUDIT), and first-degree relatives with a psychotic disorder.

After participating in the study, one patient was shown to have been misdiagnosed at the time of recruitment and did not meet the criteria for any psychotic disorder. This individual was therefore excluded from all subsequent data analysis.

### Definition of thalamic subregions

Thalamic ROIs were defined according to the following procedure: T1-images were normalized to the Montreal Neurological Institute (MNI) 152 1mm space using FSL FLIRT and FNIRT. To extract a whole thalamus ROI, the thalamus mask from the FSL Harvard-Oxford subcortical atlas was used with a 50% probability threshold. For sub-regional delineation of the thalamus, the Oxford Thalamic Connectivity Atlas ^3^ (probability thresholding at 50%) were applied to all normalized images for ROI extraction (THA-PFC, THA-TC, THA-M1, THA-PreMC and THA-PPC). The Oxford Thalamic Connectivity Atlas additionally subdivides the thalamus in the subregions THA-OC and THA SSC (with occipital and somatosensory connections, respectively). In the quantitative analysis of the subregional TACs, we were unable to get reliable estimates of [^11^C]FLB 457 BP_ND_ estimates from these regions, shown by the large standard errors around the outcome (SE > 10% of the mean BP_ND_). These two ROIs were therefore *a priori* excluded, leading to BP_ND_ from a total of six ROIs being entered into the statistical analysis (whole-thalamus and five thalamic sub-regions).

### DTI tractography

Based on the results of the PET analyses of thalamic subregions we chose to study the two major radiations leading to and from the TH-PFC ROI, the anterior thalamic radiation (ATR) and inferior thalamic radiation (ITR)^4^ . Manual dissection was done on whole brain tractography using Explore DTI, following protocols in Niida and Wang ^5,6^. For the ATR, two ROIs were placed coronally in the interior capsule, the first at the level of the genu of the corpus callosum and the second at the level of the anterior commissure ^6^. An excluding ROI was placed axially at the level of the pons in order to eliminate the frontopontine tracts. For the ITR, one ROI was placed axially covering the whole anterior temporal lobe. From a tractography based on that ROI, the ITR was selected as the only tract other than the anterior commissure directed medially toward the thalamus ^6^. If necessary to eliminate obviously aberrant tracts an exclusion ROI was placed coronally just posterior to the thalamus. See SFigure 1 for representative tractography of the ATR and ITR.

*
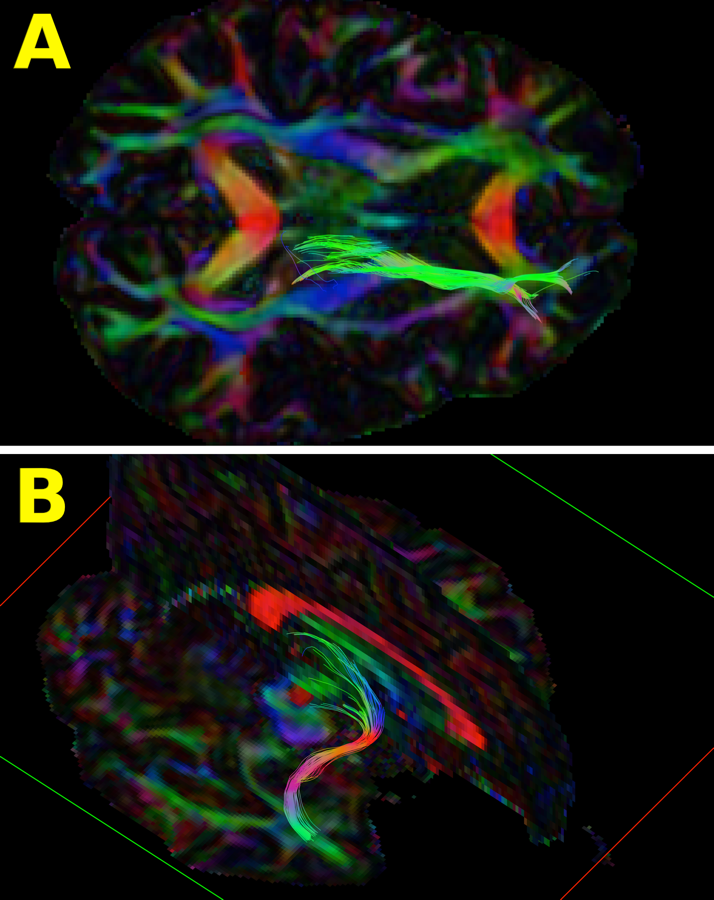
*

*sFigure 1. Tractography of the anterior (A) and inferior thalamic radiation (B) in one representative subject using conventional DTI color coding.*

### PET data acquisition and quantification

Individualized plaster helmets were made and used as head fixation devices to minimize head movement ^7^. A transmission scan with a ^137^Cs source was performed prior to the emission scan to correct for attenuation.

PET images were reconstructed into 3x1min, 4x2min and 12x6min subsequent frames using ordered subset expectation maximization. Head movement was further corrected for by frame-to-frame realignment, as described previously^8^. T1 images were co-registered to summation PET images using SPM5 (Wellcome Trust Centre for Neuroimaging, London, UK) in Matlab 2014 (The Mathworks, Natick, MA, USA). Thalamus and cerebellum ROIs were then back-transformed using MRI- PET coregistration parameters and applied to the PET data. From this, regional time activity curves (TACs) were extracted and used in the quantitative analysis.

Two patients were allowed to leave the head holding system during frame 14 and frames 7-9, respectively, due to experiencing discomfort. For both patients, a new transmission scan was then obtained for subsequent frames and the missing frames were excluded from the ensuing quantitative analysis. For each of the patient’s matched control subject, the same frames as the ones missing for the patients were discarded, to ensure the closest possible matching.

One patient did not participate in the MRI examination due to a combination of technical reasons and high symptom levels. To include data from this patient, the following procedure was applied for a PET-based ROI delineation and TAC extraction: the summation PET images from all other patients and control subjects in the study were co-registered to their corresponding T1-images. The resulting PET images were then warped into MNI space, using the transformation matrices obtained by FLIRT and FNIRT normalization of the T1-images, and averaged to form an MNI PET template. The summation PET image of the subject with missing MRI data was then normalized to this MNI PET template using FSL FLIRT and FNIRT. ROIs were extracted and warped back to individual PET space and used in the ensuing image analysis. Importantly, the T1-image of the patient’s matched control subject was discarded for this analysis and the same “PET-only” ROI delineation procedure was applied for this control, to ensure the closest possible matching.

### Statistical analysis

***Patient control matching***

We examined the matching between patients and healthy controls, by assessing differences in age, body mass index, education, injected dose of radioligand, specific radioactivity, injected mass and whole-thalamus ROI volume, using two-tailed paired and two-sample t-test ($\alpha=0.05$, BF H1 prior $\sim Cauchy(0,0.707)$, H0 prior = 0). We also calculated the cerebellum standardized uptake values (SUVs), by dividing the radioactivity in the reference region mask with injected activity and body weight. We then compared the total cerebellum SUV and SUV spanning from 25-85 minutes between patient and controls.

### *Objective 1: Analysis of whole thalamus*

The first objective of this study was to replicate the aggregate effect size from previous literature on thalamic D2-R availability differences between patients with psychotic disorders and healthy controls. Towards this end, we performed a systematic literature search on PubMed with the aim of finding all previously published PET studies using a D2-R radioligand to examine patient-control difference in the thalamus. The inclusion and exclusion criteria of studies were the same as reported in Kambeitz et al. (2014)^9^. In brief: the study needed to employ PET or SPECT performed using a radioligand that show affinity for the D2-R, including healthy controls and drug-free or drug-naïve patients with psychotic disorders or schizophrenia, with no current neurological or other psychiatric conditions. The search was restricted to the time following the search performed by Kambeitz et al. (January 1^st^ 2013) up until the March 16^th^ 2021. The following MESH search terms were used:

((("psychotic disorders"[MeSH Terms] OR ("psychotic"[All Fields] AND "disorders"[All Fields]) OR "psychotic disorders"[All Fields] OR "psychosis"[All Fields]) OR ("psychotic disorders"[MeSH Terms] OR ("psychotic"[All Fields] AND "disorders"[All Fields]) OR "psychotic disorders"[All Fields] OR ("psychotic"[All Fields] AND "disorder"[All Fields]) OR "psychotic disorder"[All Fields]) OR ("schizophrenia"[MeSH Terms] OR "schizophrenia"[All Fields])) AND (("receptors, dopamine d2"[MeSH Terms] OR ("receptors"[All Fields] AND "dopamine"[All Fields] AND "d2"[All Fields]) OR "dopamine d2 receptors"[All Fields] OR ("dopamine"[All Fields] AND "d2"[All Fields] AND "receptor"[All Fields]) OR "dopamine d2 receptor"[All Fields]) OR (DA[All Fields] AND D2R[All Fields]) OR D2R[All Fields] OR D2-R[All Fields] OR (D2[All Fields] AND availability[All Fields]) OR ("fallypride"[Supplementary Concept] OR "fallypride"[All Fields]) OR ("raclopride"[MeSH Terms] OR "raclopride"[All Fields]) OR flb457[All Fields] OR PHNO[All Fields] OR ("epidepride"[Supplementary Concept] OR "epidepride"[All Fields]))) AND (("positron-emission tomography"[MeSH Terms] OR ("positron-emission"[All Fields] AND "tomography"[All Fields]) OR "positron-emission tomography"[All Fields] OR ("positron"[All Fields] AND "emission"[All Fields] AND "tomography"[All Fields]) OR "positron emission tomography"[All Fields]) OR PET[All Fields] OR ("tomography, emission-computed, single-photon"[MeSH Terms] OR ("tomography"[All Fields] AND "emission-computed"[All Fields] AND "single-photon"[All Fields]) OR "single-photon emission-computed tomography"[All Fields] OR "spect"[All Fields]) OR ("tomography, emission-computed, single-photon"[MeSH Terms] OR ("tomography"[All Fields] AND "emission-computed"[All Fields] AND "single-photon"[All Fields]) OR "single-photon emission-computed tomography"[All Fields] OR ("single"[All Fields] AND "photon"[All Fields] AND "emission"[All Fields] AND "tomography"[All Fields]) OR "single photon emission tomography"[All Fields])) AND 2013/01/01[EDAT] : 2021/04/16[EDAT]

The search resulted in 167 articles. The abstracts were downloaded and independently coded by the first and last author of this study (PPS and SC). Following this, 43 articles were then downloaded in full, and two studies were independently deemed to report data that fulfilled the inclusion criteria ^10,11^. Reported mean binding potential and SD for patients and controls were extracted and effect sizes and their corresponding SE were calculated and inserted into a random effect meta-analysis model together with all studies reported in Kambeitz et al., 2014^9^. In addition, data from Talvik et al. (2003)^12^ , Yasuno et al. (2004) ^13^ and Veselinovic et al. (2018) ^11^ were also included. Veselinovic et al. (2018) reported relevant data after the publication of the meta-analysis by Kambeitz et al., 2014. Talvik et al. (2003) and Yasuno et al. (2004) were excluded from the analysis performed by Kambeitz et al. (2014) due to an overlapping sample with the included Talvik et al. (2006)^14^ and Suhara et al. (2003)^15^ respectively. Here, we included both Talvik et al. (2003) and Yasuno et al. (2004) results by employing a random effect meta-analysis using a variance-covariance matrix that adjusts for the correlation between the overlapping effect sizes ^16^. The ensuing overall effect size was a Cohen’s d = -0.26, with a 95% CI spanning from -0.53 to 0.00 (see SFigure 2 below).


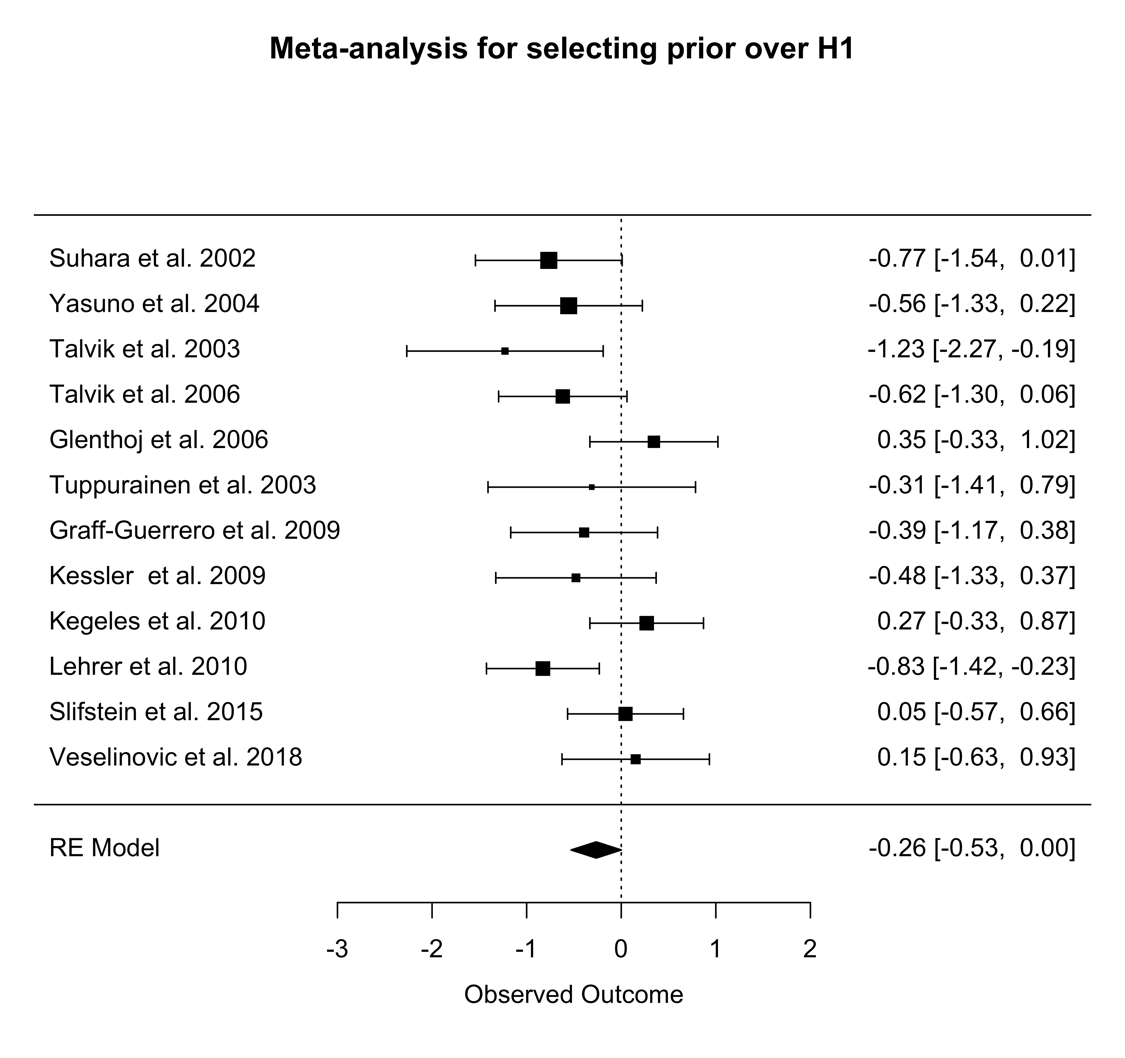


*sFigure 2. Previously published studies on D2-R availability differences between patients with psychotic disorders and healthy controls indicate that patients have lower binding in the thalamus. The partial sample overlap between Suhara et al., 2002 and Yasuno et al., 2004 as well as Talvik et al., 2003 and Talvik et al., 2006 have been accounted for in this model. The ensuing overall effect size was then used to shape the alternative hypothesis of thalamic D2-R patient-control differences the current study.*

We translated the overall effect seen in SFigure 2 into an alternative hypothesis for our study, by specifying a prior over H1 as a Normal distribution centered around -0.26 with and SD of 0.14. Since the anatomical location of previous findings in the thalamus has varied, we selected [^11^C]FLB 457 BP_ND_ in the whole-thalamus ROI as the dependent variable for this analysis.

A Bayesian paired-sample t-test was then performed to examine the evidence in favor of the this aggregated effect size on D2-R patient-control differences in thalamus against the null-hypothesis of no difference ^17,18^. By using Bayes Factor, which is a measure of relative support in data for one hypothesis over another, the evidence in favor of the alternative hypothesis (H1) over the null hypothesis (H0) can be assessed ^17,19^. A robustness-check over the alternative hypothesis is presented below.

A frequentist t-test was also performed to examine patient – control differences in [^11^C]FLB457 BP_ND_ in the whole-thalamus ($\alpha$=0.05). Although the patient-control D2-R availability difference in thalamus was not significant in the metaanalysis by Kambeitz et al.^9^ the effect size and 95% confidence interval (CI) from this model and our own meta-analyses presented in SFigure 2, indicates lower binding in patients. Hence, the frequentist paired-sample test was *a priori* specified as one-sided, expecting patients to have lower [^11^C]FLB 457 BP_ND_. We also performed two-sample t-tests for the analyses of thalamic D2-R availability (see below).

### *Objective 2: Analysis of thalamic subregions*

The second objective was to examine patient-control differences in [^11^C]FLB 457 BP_ND_ in connectivity-based subregions of the thalamus. Again, both a frequentist and equivalent Bayesian statistical test was performed.

For the Bayesian approach, paired-sample t-tests were performed. BF was then employed to assess support in the data for the alternative hypothesis (lower [^11^C]FLB 457 BP_ND_ in patients denoted as a folded normal distribution centered around 0 with and SD = 0.5) against the null hypothesis (no patient-control difference). These uninformative alternative hypotheses were selected *a priori* as this corresponds to a reasonable expected effect size of Cohen’s dz=0.5 of decreased D2-R availability in a sub-region of the thalamus ^9^. Robustness checks, with varying prior widths, were also performed (see below).

For the frequentist approach, paired-sample t-tests were carried out for each ROI. To correct for multiple comparisons, a permutation procedure was employed ^20^ to account for the dependency between the five DVs, resulting in a family-wise error corrected significance-level of 0.0356. This procedure hence allows us to estimate a correction that results in an (average) alpha of 5%, while maintaining as low an (average) beta as possible. Hence, we can maximize power, while keeping the conventional Neyman-Pearson NHST rate for type I errors.

All paired-sample t-test (frequentist and Bayesian) carried out for objective 2 were *a priori* determined to be one-sided, expecting patients to have lower [^11^C]FLB 457 BP_ND_ compared to controls (see the preregistration document at <https://osf.io/nhr3w/>).

### *Objective 3: DTI analysis*

The third objective was to examine alterations in structural connectivity between patients and control subjects in the anterior and inferior thalamic radiation. In analogy with the PET statistics no lateralization hypothesis was present and FA of right and left radiations were averaged. The literature has shown mainly an increase of FA in psychosis and schizophrenia, but not solely ^21,22^, and thus a two sided t-test was employed. Since DTI data was only available for a subset of the subjects, no matching as in the PET analyses was possible and the analysis was unpaired.

## Results

## Matching of subjects

Patients and controls did not significantly differ in age, education, body-mass index or injected radioactivity of [^11^C]FLB457. However, there was a difference between groups in both molar activity and injected mass (sTable 1).

There were no significant differences in thalamus volumes or in cerebellum SUVs between patients and healthy controls (sTable 1 and 2). BF showed support in favor of the null hypothesis of no difference for thalamus volumes, whereas for cerebellum SUVs the null hypothesis was only supported 2.8 times more by the data, compared to that of a patient-control difference of large magnitude (Cauchy(0,0.707).

For the subjects included in the DTI analysis, the demographic variables did not differ significantly between groups (gender: patients 7/4 males/females v.s. control subjects 9/6); age: 29.2 v.s. 30.4 (p=0.61); BMI: 23.4 v.s. 25.2 (p=0.17); education years 16 v.s. 15.5 (p=0.67).


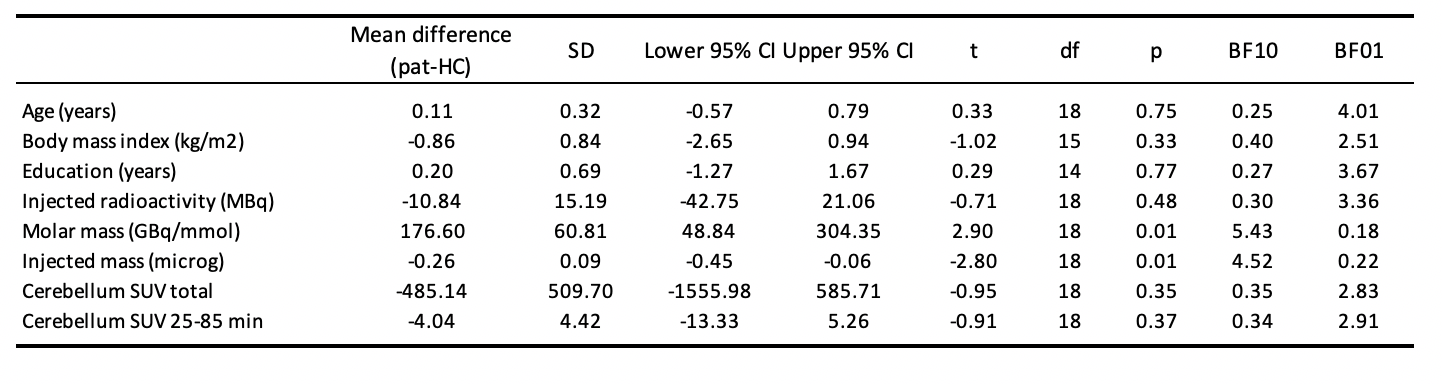
*sTable 1: Matching between patients and healthy controls was examined using frequentist and Bayes Factor (BF) paired t-test (prior over H1 Cauchy(0, 0.707). BF10: support in data in favor of the alternative hypothesis over the null hypothesis BF01: support in data in favor of the null hypothesis over the alternative hypothesis.*


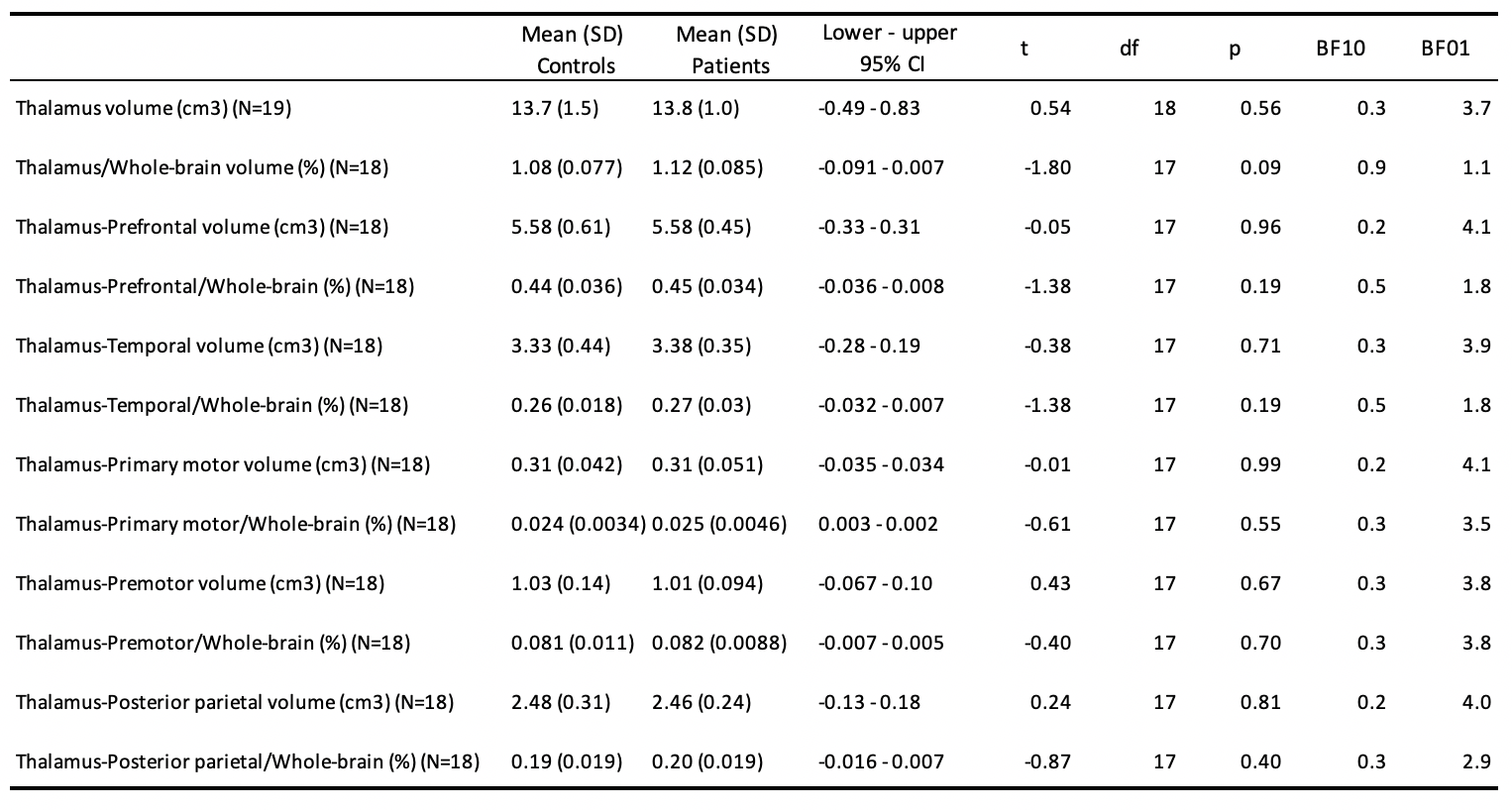


*sTable 2: Descriptive data and group comparison of patient and healthy controls volume in the whole thalamus ROI and thalamic subregions, with and without normalisation to whole-brain volume, examined using frequentist and Bayes Factor (BF) paired t-test (prior over H1 Cauchy(0, 0.707). BF10: support in data in favor of the alternative hypothesis over the null hypothesis BF01: support in data in favor of the null hypothesis over the alternative hypothesis.*

**Results according to the verbatim pre-registration**

In the pre-registration, it was stated that the alternative hypothesis for the BF t-test of the patient-control difference in the whole-thalamus would be based on the meta-analytical results from Kambeitz et al., 2014 ^9^, resulting in a gaussian distribution centered around -0.32 with an SD of 0.18. The BF in the current study in favor of a patient-control difference using this a prior was 5.14, indicating over five times more evidence in favor of a difference compared to that of no difference.

**Robustness checks**

Two-sample (unpaired) t-tests showed similar results as in the main analysis using paired t-tests (sTable2).

| **Region** | **t** | **df** | **p** | **Mean diff** | **95% CI**  **lower** | **95% CI upper** | **Cohen’s**  **d** |
| --- | --- | --- | --- | --- | --- | --- | --- |
| Whole Thalamus | 1.77 | 31.2 | 0.043 | 0.38 | -0.06 | 0.82 | 0.58 |
| Thalamus-Prefrontal Cx | 1.95 | 32.0 | 0.030 | 0.48 | -0.02 | 0.98 | 0.63 |
| Thalamus-Temporal Cx | 1.07 | 31.1 | 0.146 | 0.24 | -0.21 | 0.69 | 0.35 |
| Thalamus-Primary motor Cx | 1.51 | 30.5 | 0.071 | 0.22 | -0.08 | 0.52 | 0.49 |
| Thalamus-Premotor Cx | 0.24 | 29.7 | 0.405 | 0.04 | -0.31 | 0.39 | 0.08 |
| Thalamus-Posterior parietal Cx | 0.16 | 23.4 | 0.435 | 0.02 | -0.27 | 0.31 | 0.05 |

*sTable 3. Results from thalamic patient-control differences in [^11^C]FLB457 BP_ND_ when using two-sample (unpaired) Welch’s t-tests.*

*Patient-control difference in molar activity and injected mass*

To ascertain that any potential patient-control differences in D2-R availability were not due to differences in radiochemistry we correlated molar activity and injected mass with [^11^C]FLB 457 BP_ND_ in the whole thalamus ROI, finding no significant association between the variables (Specific activity *r* = -0.11, p = 0.53 BF_H0:H1_ = 3.84; Injected mass *r* = 0.09, p = 0.58, BF_H0:H1_ = 4.3, with BF tests using a flat prior over H1).

*Exclusion of participants*

In this study, we were 1) unable to obtain an MR for one patient, 2) one control subject had a 1.5T scan instead of 3T scan and 3) two patients briefly went out of the PET system due to experiencing discomfort (see main text). sTable 3 below presents the focal results of the study when excluding these 4 participants. The ensuing loss of power likely weakens the overall inferential statistics outcomes, however the observed effect sizes remains similar to the full sample size. It is hence unlikely that the results from these 4 subjects account for the observed patient-control difference observed in the full sample.

|  | | | | |
| --- | --- | --- | --- | --- |
| **Region** | **t** | **df** | **p** | **Cohen’s d** |
| Whole Thalamus | 1.66 | 27.7 | 0.06 | -0.57 |
| Thalamus-Prefrontal Cx | 1.75 | 27.7 | 0.05 | -0.61 |
| Thalamus-Temporal Cx | 0.82 | 26.7 | 0.21 | -0.29 |
| Thalamus-Primary motor Cx | 1.01 | 24.6 | 0.16 | -0.35 |
| Thalamus-Premotor Cx | -0.06 | 25.0 | 0.52 | 0.02 |
| Thalamus-Posterior parietal Cx | -0.06 | 19.0 | 0.53 | 0.02 |

*sTable 4. Patient-control difference in [^11^C]FLB457 BPND when excluding 4 participants.*

A robustness check over the alternative hypothesis for the Bayesian paired t-test was performed for both objective 1 and 2. We defined H1 based on all previous literature reporting on thalamic D2-R availability difference between drug-free patients with psychotic disorders and healthy controls, but only included studies that used [^18^F]Fallypride or [^11^C]FLB457, resulting in a H1: d∼N(-0.34,0.18) (an overall effect size of -0.34 with an 95% CI ranging from -0.70 to 0.02). There was still support for H1 over H0 (BF10 > 5), indicating that patients have lower BP_ND_ in the whole thalamus ROI (see sFigure 3).


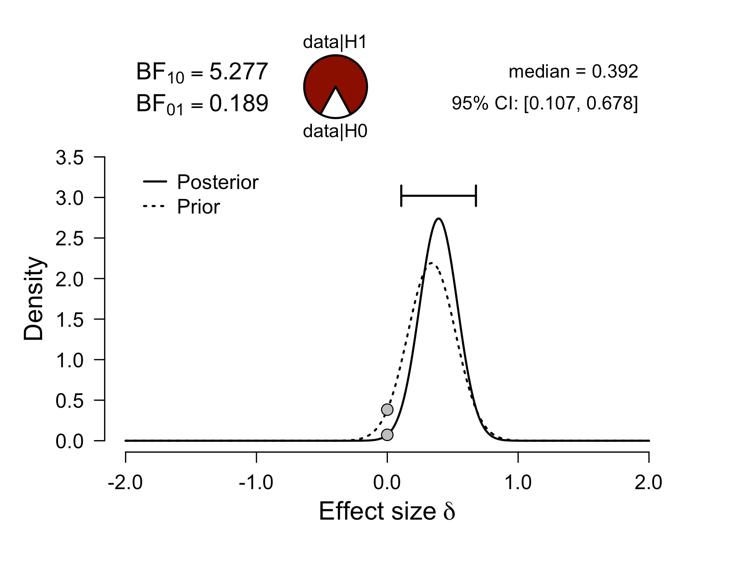


*SFigure 3. Robustness-check of the Bayesian paired-test, testing if patients have lower BP_ND_ compared to control subjects based on the alternative hypothesis generated by only including previous [^18^F]Fallypride and [^11^C]FLB457 studies on D2-R differences in the whole-thalamus between patients with psychotic disorders and healthy controls.*

When performing a robustness check using a narrow and wide (one-sided) hypothesis over the patient-control difference in [^11^C]FLB457 BP_ND_, there was still moderate support in favor of H1 in the Thalamus Prefrontal Cortex ROI, but no other ROI showed support in favor of H1. In Thalamus-Premotor and Posterior Parietal cortex, there was moderate support in favor of the null hypothesis when using a wide prior over the alternative hypothesis (sTable 5).


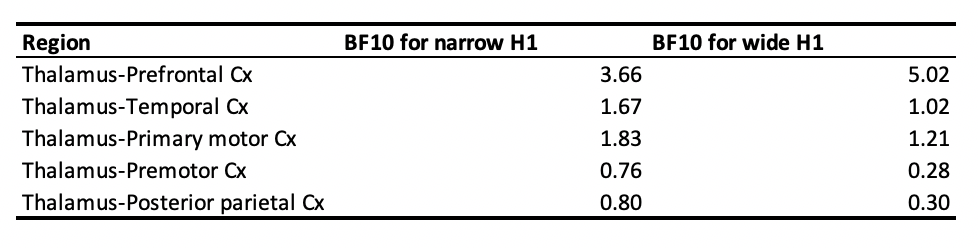


*sTable 5. Robustness-check for subregional analysis of thalamic [^11^C]FLB457 BP_ND_ differences between healthy controls and patients. Narrow H_1_: Patients are expected to have lower BP_ND_ compared to controls using a folded Normal(0,0.2) as prior over H_1_. This means that H_1_ denotes an effect described by a one-sided normal distribution with an SD of 0.2, corresponding to expecting a small patient-control effect size. Wide H_1_: Patients are expected to have lower BP_ND_ compared to controls using a folded Normal(0,0.8) as prior over H_1_. This means that H_1_ denotes an effect described by a one-sided normal distribution with an SD of 0.8, corresponding to expecting a large patient-control effect size.*

*Meta-analyses: funnel plots*

sFigure 4 show the funnel-plots for the two meta-analyses (see Figure 3 in main article).


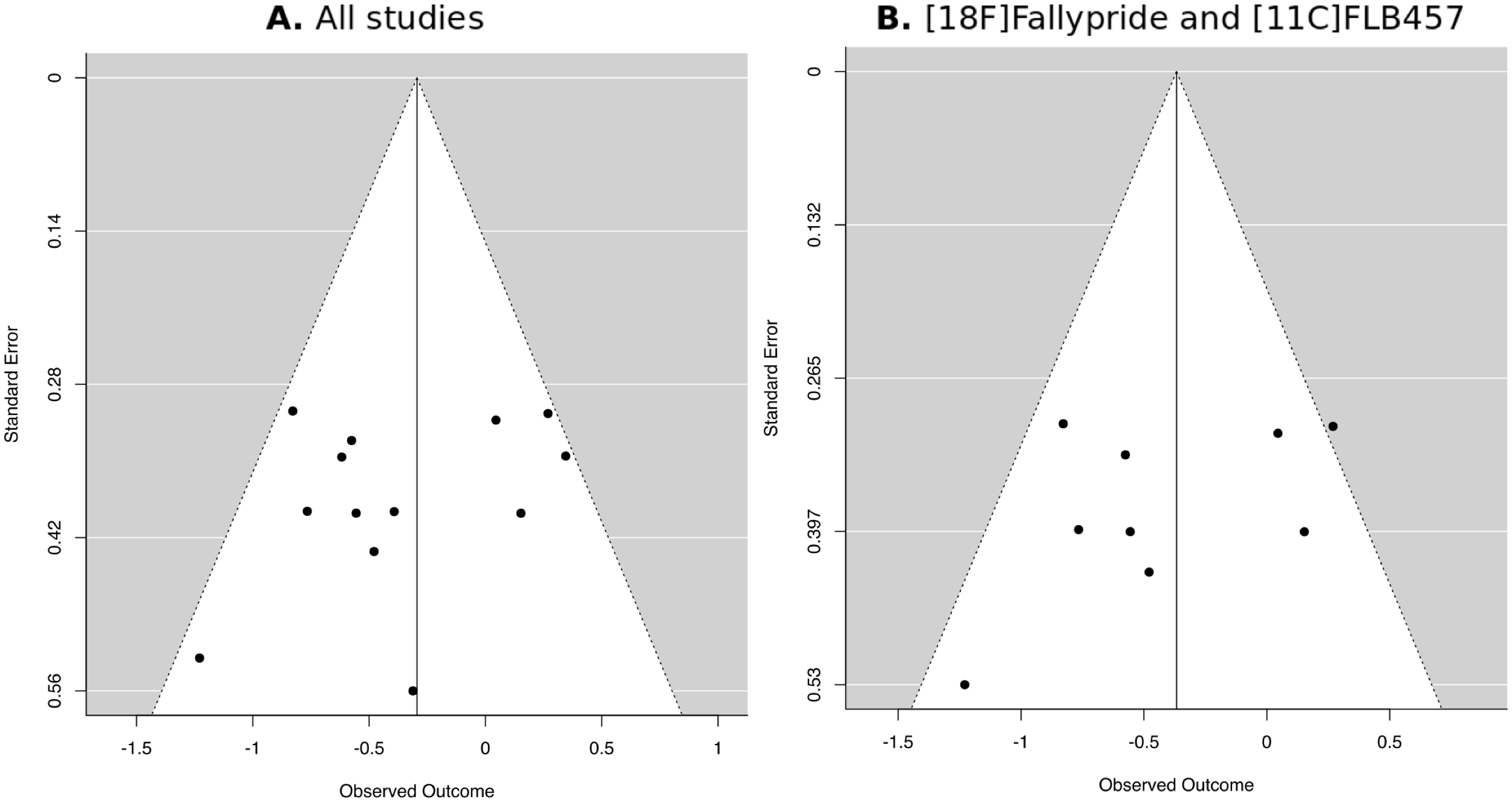


*sFigure 4. Funnel plots of the two random effect meta-analyses including data from this study. A) Including all studies. B) Including only studies using [^18^F]Fallypride or [^11^C]FLB.*

*Meta-analyses: exclusion of Talvik et al., 2006 and Yasuno et al., 2004*

All patients who participated in the Talvik et al. 2003 study ^12^ also underwent [^11^C]raclopride scans, and constituted a subsample of the patient group in Talvik et al., 2006 ^23^. A subset of patients and healthy controls in Yasuno et al., 2004^13^ constituted a subsample of the participant sample form Suhara et al., 2002^15^. in sFigure 5 show the results when fully excluding the results from Talvik et al., 2006 and Yasuno et al., 2004. The overall conclusion of significantly lower BP_ND_ in thalamus in patients is unchanged.


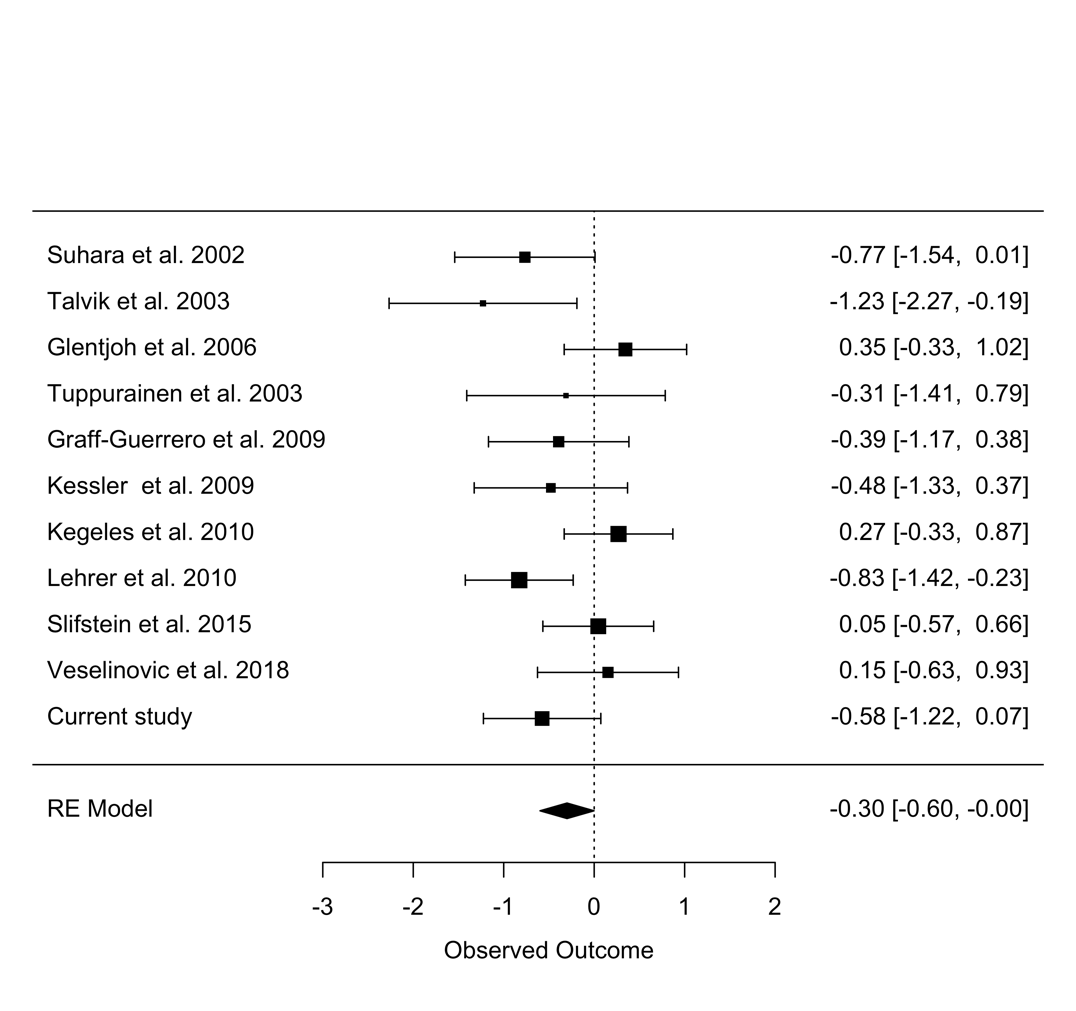


*sFigure 5. Meta-analysis excluding the data from the study Talvik et al., 2006 and Yasuno et al., 2004.*

## References

1 Saunders JB, Aasland OG, Babor TF, de la Fuente JR, Grant M. Development of the Alcohol Use Disorders Identification Test (AUDIT): WHO Collaborative Project on Early Detection of Persons with Harmful Alcohol Consumption--II. *Addiction* 1993; **88**: 791–804.

2 Berman AH, Bergman H, Palmstierna T, Schlyter F. Evaluation of the Drug Use Disorders Identifi cation Test (DUDIT) in Criminal Justice and Detoxifi cation Settings and in a Swedish Population Sample European Addiction c Re e s ar h. 2005. doi:10.1159/000081413.

3 Behrens TEJ, Johansen-Berg H, Woolrich MW, Smith SM, Wheeler-Kingshott CAM, Boulby PA *et al.* Non-invasive mapping of connections between human thalamus and cortex using diffusion imaging. *Nat Neurosci* 2003; **6**: 750–757.

4 Schmahmann JD, Pandaya DN. *Fiber pathways of the brain*. Oxford University Press: New York, 2006.

5 Niida R, Yamagata B, Niida A, Uechi A, Matsuda H, Mimura M. Aberrant anterior thalamic radiation structure in bipolar disorder: A diffusion tensor tractography study. *Front Psychiatry* 2018; **9**: 1–7.

6 Wang F, Sun T, Li XG, Liu NJ. Diffusion tensor tractography of the temporal stem on the inferior limiting sulcus: Laboratory investigation. *J Neurosurg* 2008; **108**: 775–781.

7 Bergström M, Boëthius J, Eriksson L, Greitz T, Ribbe T, Widén L. Head fixation device for reproducible position alignment in transmission CT and positron emission tomography. *J Comput Assist Tomogr* 1981; **5**: 136–141.

8 Schain M, Tóth M, Cselényi Z, Stenkrona P, Halldin C, Farde L *et al.* Quantification of serotonin transporter availability with [11C]MADAM--a comparison between the ECAT HRRT and HR systems. *Neuroimage* 2012; **60**: 800–7.

9 Kambeitz J, Abi-Dargham A, Kapur S, Howes OD. Alterations in cortical and extrastriatal subcortical dopamine function in schizophrenia: Systematic review and meta-analysis of imaging studies. *Br J Psychiatry* 2014; **204**: 420–429.

10 Slifstein M, van de Giessen E, Van Snellenberg J, Thompson JL, Narendran R, Gil R *et al.* Deficits in prefrontal cortical and extrastriatal dopamine release in schizophrenia: a positron emission tomographic functional magnetic resonance imaging study. *JAMA psychiatry* 2015; **72**: 316–24.

11 Veselinović T, Vernaleken I, Janouschek H, Cumming P, Paulzen M, Mottaghy FM *et al.* The role of striatal dopamine D 2/3 receptors in cognitive performance in drug-free patients with schizophrenia. *Psychopharmacology (Berl)* 2018; **235**: 2221–2232.

12 Talvik M, Nordström AL, Olsson H, Halldin C, Farde L. Decreased thalamic D2/D3 receptor binding in drug-naive patients with schizophrenia: A PET study with [11C]FLB 457. *Int J Neuropsychopharmacol* 2003; **6**: 361–370.

13 Yasuno F, Suhara T, Okubo Y, Sudo Y, Inoue M, Ichimiya T *et al.* Low dopamine D2 receptor binding in subregions of the thalamus in schizophrenia. *Am J Psychiatry* 2004; **161**: 1016–1022.

14 Talvik M, Nordström A-L, Okubo Y, Olsson H, Borg J, Halldin C *et al.* Dopamine D2 receptor binding in drug-naïve patients with schizophrenia examined with raclopride-C11 and positron emission tomography. *Psychiatry Res* 2006; **148**: 165–73.

15 Suhara T, Okubo Y, Yasuno F, Sudo Y, Inoue M, Ichimiya T *et al.* Decreased dopamine D2 receptor binding in the anterior cingulate cortex in schizophrenia. *Arch Gen Psychiatry* 2002; **59**: 25–30.

16 Lin D-Y, Sullivan PF. Meta-analysis of genome-wide association studies with overlapping subjects. *Am J Hum Genet* 2009; **85**: 862–72.

17 Kass RE, Raftery AE. Bayes factors. *J Am Stat Assoc* 1995; **90**: 773–795.

18 Morey RD, Wagenmakers E-J. Simple relation between Bayesian order-restricted and point-null hypothesis tests. *Stat Probab Lett* 2014; **92**: 121–124.

19 Jeffreys H. *Theory of probability*. 3rd ed. Oxford University Press: Oxford, 1961.

20 Nichols TE, Holmes AP. Nichols-2001.Pdf. 2001; **25**: 1–25.

21 Kelly S, Jahanshad N, Zalesky A, Kochunov P, Agartz I, Alloza C *et al.* Widespread white matter microstructural differences in schizophrenia across 4322 individuals: Results from the ENIGMA Schizophrenia DTI Working Group. *Mol Psychiatry* 2018; **23**: 1261–1269.

22 Barth C, Lonning V, Gurholt TP, Andreassen OA, Myhre AM, Agartz I. Exploring white matter microstructure and the impact of antipsychotics in adolescent-onset psychosis. *PLoS One* 2020; **15**: e0233684.

23 Talvik M, Nordstrom A-L, Okubo Y, Olsson H, Borg J, Halldin C *et al.* Dopamine D2 receptor binding in drug-naive patients with schizophrenia examined with raclopride-C11 and positron emission tomography. *Psychiatry Res* 2006; **148**: 165–173.
